# Supplementary material for: Factors associated with psychological symptoms in hospital workers of a French hospital during the COVID-19 pandemic: Lessons from the first wave
Source: PLoS One. 2022 Apr 28;17(4):e0267032. doi: 10.1371/journal.pone.0267032 (PMC9049512; doi:10.1371/journal.pone.0267032)
Supplement: S1 Appendix — (DOCX) [file pone.0267032.s001.docx]

**S1 Appendix: submitted questionnaire for the survey**

1. What is the location of your professional practice

- Hospital Paris Saint-Joseph
- Hospital Marie-Lannelongue

1. Did you work between March 15^th^ and May 15^th^?

- Yes
- No

If no : end of the questions.

1. Are you

- A woman
- A man

1. How old are you ?

- 18-25 ans
- 26-33 years
- 34-41 years
- 42-50 years
- 50-60 years
- + 60 years

1. What is your profession ?

- Caregiver (nurse, assistant nurse, nurse manager)
- Administrative healthcare
- Medical professional (physician, pharmacologist, biologist)
- Other caregivers (Physiotherapist, stretcher-bearer, Radiologic Technologist, Psychologist)
- Midwife
- Others

1. How long are you working in the same hospital service (years)

- <5
- ≥5

1. How long are you are you a graduate (years)

- <5
- ≥5

1. Do you have a history of professional burn-out or depression

- No
- Yes

1. What was your working area during COVID-19 crisis (N=780)

- COVID Area
- Non-COVID Area
- Other professional activity
- Remote work

1. Did you manage COVID-19 patients

- Frequently (every working day)
- Regularly (at least once a week)
- Rarely (less than once a week)
- Never

1. Have you been infected or did you have colleagues or relatives infected?

- Yes
- No

1. What is your marital situation (N=747)

- Single
- As a couple

1. What is your familial situation (N=471)

- No child
- One or several children

1. Has the COVID-19 crisis made you anxious?

- Yes
- No

1. What for were you anxious?

- Family
- Oneself
- In one’s work
- The others

1. Were you afraid of contaminating relatives?

- Yes
- No

1. Are you actually afraid of being infected?

- Yes
- No

1. Actually, do you feel good at work?

- Yes
- No

**Post-traumatic Stress Distress Checklist: PCL**

Instructions: Below is a list of problems that people sometimes have in response to a very stressful experience. Here we consider the Covid-19 crisis as a stressful event.

Please read each problem carefully and then circle one of the numbers to the right to indicate how much you have been bothered by that problem in the past month.

0 Not at all 1 A little bit 2 Moderately 3 Quite a bit 4 Extremely

1. Repeated, disturbing memories, thoughts, or images of the stressful experience?

2. Repeated, disturbing dreams of the stressful experience?

3. Suddenly acting or feeling as if the stressful experience were happening again (as if you were reliving it)?

4. Feeling very upset when something reminded you of the stressful experience?

5. Having physical reactions (e.g., heart pounding, trouble breathing, sweating) when something reminded you of the stressful experience?

6. Avoiding thinking about or talking about the stressful experience or avoiding having feelings related to it?

7. Avoiding activities or situations because they reminded you of the stressful experience?

8. Trouble remembering important parts of the stressful experience?

9. Loss of interest in activities that you used to enjoy?

10. Feeling distant or cut off from other people?

11. Feeling emotionally numb or being unable to have loving feelings for those close to you?

12. Feeling as if your future will somehow be cut short?

13. Trouble falling or staying asleep?

14. Feeling irritable or having angry outbursts?

15. Having difficulty concentrating?

16. Being "super-alert" or watchful or on guard?

17. Feeling jumpy or easily startled?

Hospital Anxiety and Depression Scale (HADS)

**Tick the box beside the reply that is closest to how you have been feeling in the past week.**

**Don’t take too long over you replies: your immediate is best.**

| **D** | **A** |  | **D** | **A** |  |
| --- | --- | --- | --- | --- | --- |
|  |  | **I feel tense or 'wound up':** |  |  | **I feel as if I am slowed down:** |
|  | 3 | Most of the time | 3 |  | Nearly all the time |
|  | 2 | A lot of the time | 2 |  | Very often |
|  | 1 | From time to time, occasionally | 1 |  | Sometimes |
|  | 0 | Not at all | 0 |  | Not at all |
|  |  |  |  |  |  |
|  |  | **I still enjoy the things I used to enjoy:** |  |  | **I get a sort of frightened feeling like 'butterflies' in the stomach:** |
| 0 |  | Definitely as much |  | 0 | Not at all |
| 1 |  | Not quite so much |  | 1 | Occasionally |
| 2 |  | Only a little |  | 2 | Quite Often |
| 3 |  | Hardly at all |  | 3 | Very Often |
|  |  |  |  |  |  |
|  |  | **I get a sort of frightened feeling as if something awful is about to**  **happen:** |  |  | **I have lost interest in my appearance:** |
|  | 3 | Very definitely and quite badly | 3 |  | Definitely |
|  | 2 | Yes, but not too badly | 2 |  | I don't take as much care as I should |
|  | 1 | A little, but it doesn't worry me | 1 |  | I may not take quite as much care |
|  | 0 | Not at all | 0 |  | I take just as much care as ever |
|  |  |  |  |  |  |
|  |  | **I can laugh and see the funny side**  **of things:** |  |  | **I feel restless as I have to be on the**  **move:** |
| 0 |  | As much as I always could |  | 3 | Very much indeed |
| 1 |  | Not quite so much now |  | 2 | Quite a lot |
| 2 |  | Definitely not so much now |  | 1 | Not very much |
| 3 |  | Not at all |  | 0 | Not at all |
|  |  | **Worrying thoughts go through my**  **mind:** |  |  | **I look forward with enjoyment to**  **things:** |
|  | 3 | A great deal of the time | 0 |  | As much as I ever did |
|  | 2 | A lot of the time | 1 |  | Rather less than I used to |
|  | 1 | From time to time, but not too often | 2 |  | Definitely less than I used to |
|  | 0 | Only occasionally | 3 |  | Hardly at all |
|  |  |  |  |  |  |
|  |  | **I feel cheerful:** |  |  | **I get sudden feelings of panic:** |
| 3 |  | Not at all |  | 3 | Very often indeed |
| 2 |  | Not often |  | 2 | Quite often |
| 1 |  | Sometimes |  | 1 | Not very often |
| 0 |  | Most of the time |  | 0 | Not at all |
|  |  |  |  |  |  |
|  |  | **I can sit at ease and feel relaxed:** |  |  | **I can enjoy a good book or radio or TV**  **program:** |
|  | 0 | Definitely | 0 |  | Often |
|  | 1 | Usually | 1 |  | Sometimes |
|  | 2 | Not Often | 2 |  | Not often |
|  | 3 | Not at all | 3 |  | Very seldom |

Please check you have answered all the questions

Scoring:

Total score: Depression (D)

0-7 = Normal

8-10 = Borderline abnormal (borderline case)

11-21 = Abnormal (case)

Total score: Anxiety (A)

0-7 = Normal

8-10 = Borderline abnormal (borderline case)

11-21 = Abnormal (case)
