# Supplementary material for: Factors associated with psychological symptoms in hospital workers of a French hospital during the COVID-19 pandemic: Lessons from the first wave
Source: PLoS One. 2022 Apr 28;17(4):e0267032. doi: 10.1371/journal.pone.0267032 (PMC9049512; doi:10.1371/journal.pone.0267032)
Supplement: S1 Table — (DOCX) [file pone.0267032.s002.docx]

**S1 Table: Univariable analysis of factors associated with the presence of emotional symptoms**

S1A Table: Univariable analysis of factors associated with the presence of symptoms of anxiety

|  | Total (N=780) | Respondents without  symptoms of anxiety (N=462) | Respondents with symptoms of anxiety (N=318) | OR [IC95%] | p-value |
| --- | --- | --- | --- | --- | --- |
| **Demographic characteristics** | | | | | |
| Gender | | | |  |  |
| Men | 142 (18.2%) | 102 (22.1%) | 40 (12.6%) | 1.00 | 0.001 |
| Women | 638 (81.8%) | 360 (77.9%) | 278 (87.4%) | 1.97 [1.32;2.93] |  |
| Age (years) | | | |  |  |
| ≤41 | 429 (55.0%) | 249 (53.9%) | 180 (56.6%) | 1.00 | 0.45 |
| >41 | 351 (45.0%) | 213 (46.1%) | 138 (43.4%) | 0.90 [0.67;1.19] |  |
| Marital situation (N=747) | | | |  |  |
| Single | 235 (31.5%) | 129 (29.2%) | 106 (34.6%) | 1.00 | 0.12 |
| As a couple | 512 (68.5%) | 312 (70.8%) | 200 (65.4%) | 0.78 [0.57;1.07] |  |
| Familial situation (N=471) | | | |  |  |
| No child | 72 (15.3%) | 42 (15.3%) | 30 (15.2%) | 1.00 | 0.98 |
| One or several children | 399 (84.7%) | 232 (84.7%) | 167 (84.8%) | 1.01 [0.61;1.68] |  |
| **COVID-19 professional experience** | | | | | |
| COVID-Unit assignment | | | |  |  |
| No | 405 (51.9%) | 229 (49.6%) | 176 (55.3%) | 1.00 | 0.11 |
| Yes | 375 (48.1%) | 233 (50.4%) | 142 (44.7%) | 0.79 [0.60;1.06] |  |
| Non COVID-Unit assignment | | | |  |  |
| No | 433 (55.5%) | 255 (55.2%) | 178 (56.0%) | 1.00 | 0.83 |
| Yes | 347 (44.5%) | 207 (44.8%) | 140 (44.0%) | 0.97 [0.73;1.29] |  |
| Remote work | | | |  |  |
| No | 675 (86.5%) | 407 (88.1%) | 268 (84.3%) | 1.00 | 0.12 |
| Yes | 105 (13.5%) | 55 (11.9%) | 50 (15.7%) | 1.38 [0.91;2.09] |  |
| Non-clinical professional activity | | | |  |  |
| No | 637 (81.7%) | 378 (81.8%) | 259 (81.5%) | 1.00 | 0.89 |
| Yes | 143 (18.3%) | 84 (18.2%) | 59 (18.5%) | 1.03 [0.71;1.48] |  |
| Covid-19 patients management | | | |  |  |
| Never / Rarely | 419 (53.7%) | 237 (51.3%) | 182 (57.2%) | 1.00 | 0.102 |
| Regularly / Frequently | 361 (46.3%) | 225 (48.7%) | 136 (42.8%) | 0.79 [0.59;1.05] |  |
| Respondents who were infected or having infected colleagues or relatives | | | |  |  |
| No | 314 (40.3%) | 211 (45.7%) | 103 (32.4%) | 1.00 | <0.001 |
| Yes | 466 (59.7%) | 251 (54.3%) | 215 (67.6%) | 1.75 [1.30;2.36] |  |
| **Profession** | | | |  |  |
| Administrative healthcare | 141 (18.1%) | 76 (16.5%) | 65 (20.4%) | 1.00 | 0.033 |
| Medical professional | 125 (16.0%) | 90 (19.5%) | 35 (11.0%) | 0.45 [0.27;0.76] |  |
| Caregiver | 367 (47.0%) | 209 (45.2%) | 158 (49.7%) | 0.88 [0.6;1.31] |  |
| Other Caregiver | 72 (9.2%) | 44 (9.5%) | 28 (8.8%) | 0.74 [0.42;1.33] |  |
| Midwife | 21 (2.7%) | 14 (3.0%) | 7 (2.2%) | 0.58 [0.22;1.54] |  |
| others | 54 (6.9%) | 29 (6.3%) | 25 (7.9%) | 1.01 [0.54;1.89] |  |
| Place of professional practice | | | |  |  |
| Hospital Paris Saint-Joseph | 675 (86.5%) | 399 (86.4%) | 276 (86.8%) | 1.00 | 0.86 |
| Hospital Marie-Lannelongue | 105 (13.5%) | 63 (13.6%) | 42 (13.2%) | 0.96 [0.63;1.47] |  |
| Professional experience in the same hospital service (years) | | | |  |  |
| <5 | 437 (56.0%) | 256 (55.4%) | 181 (56.9%) | 1.00 | 0.68 |
| ≥5 | 343 (44.0%) | 206 (44.6%) | 137 (43.1%) | 0.94 [0.71;1.25] |  |
| **COVID-19 emotional experience** | | | | | |
| **Anxiety during 1^st^ Wave** | | | |  |  |
| No | 294 (37.7%) | 248 (53.7%) | 46 (14.5%) | 1.00 | <0.001 |
| Yes | 486 (62.3%) | 214 (46.3%) | 272 (85.5%) | 6.85 [4.77;9.84] |  |
| Anxiety for oneself (N=486) | | | |  |  |
| No | 207 (42.6%) | 96 (44.9%) | 111 (40.8%) | 1.00 | 0.37 |
| Yes | 279 (57.4%) | 118 (55.1%) | 161 (59.2%) | 1.18 [0.82;1.69] |  |
| Anxiety for family (N=486) | | | |  |  |
| No | 65 (13.4%) | 29 (13.5%) | 36 (13.2%) | 1.00 | 0.92 |
| Yes | 421 (86.6%) | 185 (86.5%) | 236 (86.8%) | 1.03 [0.61;1.74] |  |
| Anxiety for others (N=486) | | | |  |  |
| No | 280 (57.6%) | 121 (56.5%) | 159 (58.5%) | 1.00 | 0.67 |
| Yes | 206 (42.4%) | 93 (43.5%) | 113 (41.5%) | 0.92 [0.64;1.33] |  |
| Anxiety at work (N=486) | | | |  |  |
| No | 239 (49.2%) | 116 (54.2%) | 123 (45.2%) | 1.00 | 0.049 |
| Yes | 247 (50.8%) | 98 (45.8%) | 149 (54.8%) | 1.43 [1.00;2.05] |  |
| **Fear of contaminating relatives during the 1^st^ wave** | | | |  |  |
| No | 197 (25.3%) | 147 (31.8%) | 50 (15.7%) | 1.00 | <0.001 |
| Yes | 583 (74.7%) | 315 (68.2%) | 268 (84.3%) | 2.5 [1.75;3.58] |  |
|  |  |  |  |  |  |
| **Emotional experience at time of survey** | | | | | |
| Current job satisfaction | | | |  |  |
| No | 197 (25.3%) | 60 (13.0%) | 137 (43.1%) | 1.00 | <0.001 |
| Yes | 583 (74.7%) | 402 (87.0%) | 181 (56.9%) | 0.20 [0.14;0.28] |  |
| Professional burn-out or depression history | | | |  |  |
| No | 605 (77.6%) | 394 (85.3%) | 211 (66.3%) | 1.00 | <0.001 |
| Yes | 175 (22.4%) | 68 (14.7%) | 107 (33.7%) | 2.94 [2.08;4.16] |  |
| Date of depression or burn-out history (N=175) | | |  |  |  |
| <3years | 70 (40.0%) | 21 (30.9%) | 49 (45.8%) | 1.00 | 0.05 |
| >3years | 105 (60.0%) | 47 (69.1%) | 58 (54.2%) | 0.53 [0.28;1.00] |  |

S1B Table. Univariable analysis of factors associated with the presence of symptoms of depression

|  | Total  (N=780) | Respondents without  symptoms of depression(N=618) | Respondents with  symptoms of depression  (N=162) | OR [IC95%] | p-value |
| --- | --- | --- | --- | --- | --- |
| Demographic characteristics | | | | | |
| Gender | | | |  |  |
| Men | 142 (18.2%) | 114 (18.5%) | 28 (17.3%) | 1.00 | 0.73 |
| Women | 638 (81.8%) | 504 (81.5%) | 134 (82.7%) | 1.08 [0.69;1.71] |  |
| Age (years) | | | |  |  |
| ≤41 | 429 (5%) | 344 (55.7%) | 85 (52.5%) | 1.00 | 0.47 |
| >41 | 351 (5%) | 274 (44.3%) | 77 (47.5%) | 1.14 [0.80;1.61] |  |
| Marital situation (N=747) | | | |  |  |
| Single | 235 (31.5%) | 176 (29.8%) | 59 (37.8%) | 1.00 | 0.05 |
| As a couple | 512 (68.5%) | 415 (70.2%) | 97 (62.2%) | 0.70 [0.48;1.01] |  |
| Familial situation (N=471) | | | |  |  |
| No child | 72 (15.3%) | 59 (15.8%) | 13 (13.4%) | 1.00 | 0.56 |
| One or several children | 399 (84.7%) | 315 (84.2%) | 84 (86.6%) | 1.21 [0.63;2.31] |  |
| COVID-19 professional experience | | | | | |
| COVID-Unit assignment | | | |  |  |
| No | 405 (51.9%) | 308 (49.8%) | 97 (59.9%) | 1.00 | 0.023 |
| Yes | 375 (48.1%) | 310 (50.2%) | 65 (40.1%) | 0.67 [0.47;0.95] |  |
| Non COVID-Unit assignment | | | |  |  |
| No | 433 (55.5%) | 350 (56.6%) | 83 (51.2%) | 1.00 | 0.22 |
| Yes | 347 (44.5%) | 268 (43.4%) | 79 (48.8%) | 1.24 [0.88;1.76] |  |
| Remote work | | | | | |
| No | 675 (86.5%) | 543 (87.9%) | 132 (81.5%) | 1.00 | 0.034 |
| Yes | 105 (13.5%) | 75 (12.1%) | 30 (18.5%) | 1.65 [1.03;2.62] |  |
| Non-clinical professional activity | | | |  |  |
| No | 637 (81.7%) | 503 (81.4%) | 134 (82.7%) | 1.00 | 0.70 |
| Yes | 143 (18.3%) | 115 (18.6%) | 28 (17.3%) | 0.91 [0.58;1.44] |  |
| COVID-19 patients management | | | |  |  |
| Never/Rarely | 419 (53.7%) | 328 (53.1%) | 91 (56.2%) | 1.00 | 0.48 |
| Frequently/ Regularly | 361 (46.3%) | 290 (46.9%) | 71 (43.8%) | 0.88 [0.62;1.25] |  |
| Respondents who were infected or having infected colleagues or relatives | | | |  |  |
| No | 314 (40.3%) | 257 (41.6%) | 57 (35.2%) | 1.00 | 0.14 |
| Yes | 466 (59.7%) | 361 (58.4%) | 105 (64.8%) | 1.31 [0.91;1.88] |  |
| **Profession** | | | |  |  |
| Administrative healthcare | 141 (18.1%) | 103 (16.7%) | 38 (23.5%) | 1.00 | 0.17 |
| Medical professional | 125 (16.0%) | 101 (16.3%) | 24 (14.8%) | 0.64 [0.36;1.15] |  |
| Caregiver | 367 (47.0%) | 300 (48.5%) | 67 (41.4%) | 0.61 [0.38;0.96] |  |
| Other Caregiver | 72 (9.2%) | 53 (8.6%) | 19 (11.7%) | 0.97 [0.51;1.85] |  |
| Midwife | 21 (2.7%) | 19 (3.1%) | 2 (1.2%) | 0.29 [0.06;1.28] |  |
| others | 54 (6.9%) | 42 (6.8%) | 12 (7.4%) | 0.77 [0.37;1.63] |  |
| **Place of professional practice** | | | |  |  |
| Hôpital Saint-Joseph | 675 (86.5%) | 540 (87.4%) | 135 (83.3%) | 1.00 | 0.196 |
| Marie-Lannelongue | 105 (13.5%) | 78 (12.6%) | 27 (16.7%) | 1.38 [0.86;2.23] |  |
| **Professional experience in the same hospital service (years)** | | | |  |  |
| <5 | 437 (56.0%) | 351 (56.8%) | 86 (53.1%) | 1.00 | 0.40 |
| ≥5 | 343 (44.0%) | 267 (43.2%) | 76 (46.9%) | 1.16 [0.82;1.64] |  |
| COVID-19 emotional experience | | | | | |
| Anxiety during 1st Wave | | | |  |  |
| No | 294 (37.7%) | 255 (41.3%) | 39 (24.1%) | 1.00 | <0.001 |
| Yes | 486 (62.3%) | 363 (58.7%) | 123 (75.9%) | 2.22 [1.49;3.29] |  |
| Anxiety for oneself (N=486) | | | |  |  |
| No | 207 (42.6%) | 157 (43.2%) | 50 (40.6%) | 1.00 | 0.61 |
| Yes | 279 (57.4%) | 206 (56.8%) | 73 (59.4%) | 1.11 [0.73;1.69] |  |
| Anxiety for family (N=486) | | | |  |  |
| No | 65 (13.4%) | 49 (13.5%) | 16 (13.0%) | 1.00 | 0.89 |
| Yes | 421 (86.6%) | 314 (86.5%) | 107 (87.0%) | 1.04 [0.57;1.91] |  |
| Anxiety for others (N=486) | | | |  |  |
| No | 280 (57.6%) | 208 (57.3%) | 72 (58.5%) | 1.00 | 0.81 |
| Yes | 206 (42.4%) | 155 (42.7%) | 51 (41.5%) | 0.95 [0.63;1.44] |  |
| Anxiety at work (N=486) | | | |  |  |
| No | 239 (49.2%) | 178 (49.0%) | 61 (49.6%) | 1.00 | 0.91 |
| Yes | 247 (50.8%) | 185 (51.0%) | 62 (50.4%) | 0.98 [0.65;1.47] |  |
| Fear of contaminating relatives during the 1st wave | | | |  |  |
| Yes | 197 (25.3%) | 161 (26.1%) | 36 (22.2%) | 1.00 | 0.32 |
| No | 583 (74.7%) | 457 (73.9%) | 126 (77.8%) | 1.23 [0.82;1.86] |  |
| Emotional experience at time of survey | | | | | |
| Current job satisfaction | | | |  |  |
| No | 197 (25.3%) | 109 (17.6%) | 88 (54.3%) | 1.00 | <0.001 |
| Yes | 583 (74.7%) | 509 (82.4%) | 74 (45.7%) | 0.18 [0.12;0.26] |  |
| Professional burn-out or depression history | | | |  |  |
| No | 605 (77.6%) | 506 (81.9%) | 99 (61.1%) | 1.00 | <0.001 |
| Yes | 175 (22.4%) | 112 (18.1%) | 63 (38.9%) | 2.88 [1.97;4.19] |  |
| Date of depression or burn-out history (N=175) | | | |  |  |
| <3years | 70 (40.0%) | 40 (35.7%) | 30 (47.6%) | 1.00 | 0.12 |
| >3years | 105 (60.0%) | 72 (64.3%) | 33 (52.4%) | 0.61 [0.33;1.14] |  |

S1C Table. Univariable analysis of factors associated with the presence of symptoms of post-traumatic stress

|  | Total  (N=780) | Respondents without  symptoms of post-traumatic stress (N=669) | Respondents with  symptoms of post-traumatic stress (N=111) | OR [IC95%] | p |
| --- | --- | --- | --- | --- | --- |
| Demographic characteristics | | | | | |
| Gender | | | |  |  |
| Men | 142 (18.2%) | 130 (19.4%) | 12 (10.8%) | 1.00 | 0.029 |
| Women | 638 (81.8%) | 539 (80.6%) | 99 (89.2%) | 1.99 [1.06;3.73] |  |
| Age (years) | | | |  |  |
| ≤41 | 429 (55.0%) | 362 (54.1%) | 67 (60.4%) | 1.00 | 0.22 |
| >41 | 351 (45.0%) | 307 (45.9%) | 44 (39.6%) | 0.77 [0.51;1.17] |  |
| Marital situation (N=747) | | | |  |  |
| Single | 235 (31.5%) | 188 (29.4%) | 47 (43.9%) | 1.00 | 0.003 |
| As a couple | 512 (68.5) | 452 (70.6%) | 60 (56.1%) | 0.53 [0.35;0.81] |  |
| Familial situation (N=471) | | | |  |  |
| No child | 72 (15.3%) | 62 (15.2%) | 10 (15.6%) | 1.00 | 0.94 |
| One or several children | 399 (84.7%) | 345 (84.8%) | 54 (84.4%) | 0.97 [0.47;2.01] |  |
| COVID-19 professional experience | | | | | |
| **COVID-Unit assignment** | | | |  |  |
| No | 405 (51.9%) | 351 (52.5%) | 54 (48.6%) | 1.00 | 0.46 |
| Yes | 375 (48.1%) | 318 (47.5%) | 57 (51.4%) | 1.17 [0.78;1.74] |  |
| **Non COVID-Unit assignment** | | | |  |  |
| No | 433 (55.5%) | 373 (55.7%) | 60 (54.1%) | 1.00 | 0.74 |
| Yes | 347 (44.5%) | 296 (44.3%) | 51 (45.9%) | 1.07 [0.72;1.60] |  |
| **Remote work** | | | |  |  |
| No | 675 (86.5%) | 578 (86.4%) | 97 (87.4%) | 1.00 | 0.78 |
| Yes | 105 (13.5%) | 91 (13.6%) | 14 (12.6%) | 0.92 [0.50;1.67] |  |
| **Non-clinical professional activity** | | | |  |  |
| No | 637 (81.7%) | 539 (80.6%) | 98 (88.3%) | 1.00 | 0.05 |
| Yes | 143 (18.3%) | 130 (19.4%) | 13 (11.7%) | 0.55 [0.30;1.01] |  |
| **Covid-19 patients management** | | | |  |  |
| Frequently / Regularly | 419 (53.7%) | 360 (53.8%) | 59 (53.2%) | 1.00 | 0.90 |
| Never / Rarely | 361 (46.3%) | 309 (46.2%) | 52 (46.8%) | 1.03 [0.69;1.54] |  |
| **Respondents who were infected or having infected colleagues or relatives** | | | |  |  |
| No | 314 (40.3%) | 285 (42.6%) | 29 (26.1%) | 1.00 | 0.001 |
| Yes | 466 (59.7%) | 384 (57.4%) | 82 (73.9%) | 2.10 [1.34;3.29] |  |
| **Profession** | | | |  |  |
| Administrative healthcare | 141 (18.1%) | 119 (17.8%) | 22 (19.8%) | 1.00 | 0.12 |
| Medical professional | 125 (16.0%) | 115 (17.2%) | 10 (9.1%) | 0.47 [0.21;1.04] |  |
| Caregiver | 367 (47.1%) | 305 (45.6%) | 62 (55.9%) | 1.1 [0.65;1.87] |  |
| Other Caregiver | 72 (9.2%) | 65 (9.7%) | 7 (6.3%) | 0.58 [0.24;1.44] |  |
| Midwife | 21 (2.7%) | 17 (2.5%) | 4 (3.6%) | 1.27 [0.39;4.14] |  |
| others | 54 (6.9%) | 48 (7.2%) | 6 (5.4%) | 0.68 [0.26;1.77] |  |
| **Place of professional practice** | | | |  |  |
| Hôpital Saint-Joseph | 675 (86.5%) | 581 (86.8%) | 94 (84.7%) | 1.00 | 0.548 |
| Marie-Lannelongue | 105 (13.5%) | 88 (13.2%) | 17 (15.3%) | 1.19 [0.68;2.10] |  |
| **Professional experience in the same hospital service (years)** | | | |  |  |
| <5 | 437 (56.0%) | 375 (56.1%) | 62 (55.9%) | 1.00 | 0.97 |
| ≥5 | 343 (44.0%) | 294 (43.9%) | 49 (44.1%) | 1.01 [0.67;1.51] |  |
| COVID-19 emotional experience | | | | | |
| **Anxiety during 1st Wave** | | | |  |  |
| No | 294 (37.7%) | 283 (42.3%) | 11 (9.9%) | 1.00 | <0.001 |
| Yes | 486 (62.3%) | 386 (57.7%) | 100 (90.1%) | 6.67 [3.51;12.66] |  |
| Anxiety for oneself (N=486) | | | |  |  |
| No | 207 (42.6%) | 169 (43.8%) | 38 (38.0%) | 1.00 | 0.30 |
| Yes | 279 (57.4%) | 217 (56.2%) | 62 (62.0%) | 1.27 [0.81;1.99] |  |
| Anxiety for family (N=486) | | | |  |  |
| No | 65 (13.4%) | 54 (14.0%) | 11 (11.0%) | 1.00 | 0.43 |
| Yes | 421 (86.6%) | 332 (86.0%) | 89 (89.0%) | 1.32 [0.66;2.62] |  |
| Anxiety for others (N=486) | | | |  |  |
| No | 280 (57.6%) | 225 (58.3%) | 55 (55.0%) | 1.00 | 0.55 |
| Yes | 206 (42.4%) | 161 (41.7%) | 45 (45.0%) | 1.14 [0.73;1.78] |  |
| Anxiety at work (N=486) | | | |  |  |
| No | 239 (49.2%) | 202 (52.3%) | 37 (37.0%) | 1.00 | 0.006 |
| Yes | 247 (50.8%) | 184 (47.7%) | 63 (63.0%) | 1.87 [1.19;2.94] |  |
| **Fear of contaminating relatives during the 1st wave** | | | |  |  |
| No | 197 (25.3%) | 185 (27.6%) | 12 (10.8%) | 1.00 | <0.001 |
| Yes | 583 (74.7%) | 484 (72.4%) | 99 (89.2%) | 3.15 [1.69;5.88] |  |
| Emotional experience at time of survey | | | | | |
| **Current job satisfaction** | | | |  |  |
| No | 197 (25.3%) | 139 (20.8%) | 58 (52.2%) | 1.00 | <0.001 |
| Yes | 583 (74.7%) | 530 (79.2%) | 53 (47.8%) | 0.24 [0.16;0.36] |  |
| **Professional burn-out or depression history** | | | |  |  |
| No | 605 (77.6%) | 532 (79.5%) | 73 (65.8%) | 1.00 | 0.001 |
| Yes | 175 (22.4%) | 137 (20.5%) | 38 (34.2%) | 2.02 [1.31;3.12] |  |
| Date of depression or burn-out history (N=175) | | | |  |  |
| <3years | 70 (40.0%) | 52 (38.0%) | 18 (47.4%) | 1.00 | 0.29 |
| >3years | 105 (60.0%) | 85 (62.0%) | 20 (52.6%) | 0.68 [0.33;1.40] |  |
