# Supplementary material for: Factors associated with psychological symptoms in hospital workers of a French hospital during the COVID-19 pandemic: Lessons from the first wave
Source: PLoS One. 2022 Apr 28;17(4):e0267032. doi: 10.1371/journal.pone.0267032 (PMC9049512; doi:10.1371/journal.pone.0267032)
Supplement: Attachment — Submitted filename: response to reviewers.docx [file pone.0267032.s004.docx]

Dear Sir,

Thank you for your time and consideration.

We have done a lot of work to modify the text according to the referees' recommendations; the manuscript has been throughly re-read, the tables modified and clarified. We believe the document is more understandable than when first submitted.

We hope that this corrected manuscript is now suitable for publication in PlosOne.

In the following paragraphs, we have detailed all the corrections made and added some responses to the referee's comments.

Kind regards,

Dr Marguerite d’Ussel and the other authors

| **Comments** | **Answers** |
| --- | --- |
| With so many predictors an issue of overfitting is a real danger. Can you, please, report on the percentage of variance explained in the DVs (any pseudo R^2^, e.g., Nagelkerke). | We agree with the reviewer. We added the percentage of variance explained (Nagelkerke pseudo R²). |
| What about multicollinearity among the predictors? Was this analysis performed (e.g., Tabachnik and Fidell, 2013)? | As you will see on the following tables 1, 2 and 3 there is no over fitting. Moreover we use 2 by 2 tables to test relationship between variables. This lead us to suppress some variables: Professional experience linked with Professional experience in the same hospital unit, and Usual job satisfaction linked with Current job satisfaction. |
| Some decisions appear arbitrary or not elaborated properly. For example, why age variable was categorized instead of used as it is? Since you categorized it from the beginning - even in the questionnaire (i.e., it was not done post-hoc) - there must be some reasons for it, that were not elaborated enough | To elaborate the questionnaire and to decide which variable to include in it, and furthermore how to use these variables (in binarizing/categorizing them), we have studied the previous literature with attention. We have reviewed the literature about the psychological impact of previous epidemics on healthcare workers, and their associated factors. Thus, we have used the different items as they were used in this literature: age (Chong et al. 2004; Wu et al. 2009), work experience (Chong et al. 2004), job title (Chong et al. 2004), marital status(Chong et al. 2004; Lin et al. 2007; Su et al. 2007; 2007; 2007; Wu et al. 2009), past history of psychological disorder (Su et al. 2007), working units (Chong et al. 2004; Su et al. 2007)… |
| Furthermore, you decided to use the first category (age 18-25) as a referential value. I wonder why, why not the last one? Anyway, you should elaborate on it and justify other decisions in binarizing/categorizing your variables (prof. experience, job satisfaction etc.). | This is always an arbitrary choice. Usually, when a variable is ordered we choose the highest or the lowest category. As regard age we finally binarized the variable, according to the limit corresponding roughly to the median. We also considered only 2 sites (Hospital Paris Saint-Joseph and Hospital Marie-Lannelongue) because most employees working at both hospitals worked mainly in Paris Saint-Joseph. As regard profession, we chose arbitrary as baseline administrative healthcare because those persons belonging to these profession were the less expose to Covid-19. |
| Generally, why did you decide to use cut off criteria for anxiety, depression, and PTSD and binarize them instead of exploit the full amount of information in these variables as the continual ones? You had rightly mentioned as one of the limitations of your work that these were not proper diagnoses, so why did you choose to behave as they were? Please, elaborate | After discussion with psychiatric experts we have chosen the HAD scale and PCL to determine anxiety, depression and PTSD symptoms even if these scales were not considered as diagnostic scales but as screening tests: the HADS is quick to fill, has a good validity in its French translation, and can be used among any population. According to this review (Bjelland et al. 2002) it must be used by binarization with a cut off. The best advantage with the PCL is that it summarized the characteristics of latest DSM classification for PTSD. This scale is also used with a published binarized cut off (Yao et al. 2003) |
| In order to make tables more readable I suggest at least to skip unadjusted model or model 1. | In clinical epidemiology it is usual practice to present both adjusted and unadjusted OR. We therefore maintain the unadjusted model in the table.  When using a hierarchical model it is also the way to present results (Gazmararian et al. 2000) |
| Please amend your current ethics statement to include the full name of the ethics committee/institutional review board(s) that approved your specific study. | The study has been done in accordance with the Declaration of Helsinki and approved by the GERM (Groupe Ethique et Recherche Médicale/ Ethics and Medical Research Group) from the Hospital Paris Saint-Joseph (IRB number 00012157). |
| You indicated that informed consent was not necessary for your study. Could you please provide further details on why your study is exempt from the need for consent and confirmation from your institutional review board or research ethics committee (e.g., in the form of a letter or email correspondence) that ethics review was not necessary for this study? Please include a copy of the correspondence as an "Other" file. | This research is part of the institutional care for its employees. In this context, the French regulations (JORF n°0160 of 13 July 2018 text n°110, MR-004) do not require consent but require the transmission of an information note to the employees setting out the purpose of the research. The employees’ non-opposition to the use their data for research purposes is also collected in accordance with the European General Data Protection Regulation (GDPR).  Certification of ethical opinion from the institutional ethics committee is provided as a complementary document. |
| We note that you have stated that you will provide repository information for your data at acceptance. Should your manuscript be accepted for publication, we will hold it until you provide the relevant accession numbers or DOIs necessary to access your data. If you wish to make changes to your Data Availability statement, please describe these changes in your cover letter and we will update your Data Availability statement to reflect the information you provide. | Data are contained within the Supporting Information files, and available in Excel format on direct request to the first author : [mdussel@ghpsj.fr](mailto:mdussel@ghpsj.fr) |
| Please amend either the title on the online submission form (via Edit Submission) or the title in the manuscript so that they are identical | Suppression of the short title in the manuscript |
| The Table 1. and 2. are followed by the description which is redundant, so the tables could be deleted or the text should point out only important findings | The manuscript has been modified to avoid redundancies, and table 2 has been deleted |
| The major problem is the questionnaire used to collect data on socio-demographics. Its structure, type of questions (majority of items are binary and some are multiple responses), and information collected prevent us from making solid judgments and conclusions – this is extremely important as these items are major predictors in the study. Some items (especially those about COVID19 infection) are ambiguous and nothing can be deduced based on the respondents’ answers – were respondents infected or whether they infected someone else? | To elaborate the questionnaire and choose the variables, we have studied the literature about the psychological impact of previous epidemics on healthcare workers (Chong et al. 2004; Wu et al. 2009; Lin et al. 2007; Su et al. 2007). Especially for the item about COVID-19 infection, we have based on this article (Wu et al. 2009) where it was asked if the healthcare workers had been infected or had friends or relatives who had been infected |
| These should have been kept separate to allow for making conclusions. I am missing the rationale why the majority of items are categorical. For example, respondents were grouped by age in several groups, and this also applies to other variables like working experience, patient management, etc. – my advice is to use the variables as continuous instead of categorical variables. | To elaborate the questionnaire and to decide which variable to include in it, and furthermore how to use these variables (in binarizing/categorizing them), we have studied the previous literature with attention. We have reviewed the literature about the psychological impact of previous epidemics on healthcare workers, and their associated factors. Thus, we have used the different items as they were used in this literature: age (Chong et al. 2004; Wu et al. 2009), work experience (Chong et al. 2004), job title (Chong et al. 2004), marital status(Chong et al. 2004; Lin et al. 2007; Su et al. 2007; 2007; 2007; Wu et al. 2009), past history of psychological disorder (Su et al. 2007), working units (Chong et al. 2004; Su et al. 2007)… |
| Do two hospitals from which participants were recruited differ in some aspects of work or they were selected at random? Is there some reason to believe that healthcare workers would perceive and experience the pandemic differently in these two hospitals? | Although belonging to the same group, the two hospitals have different specificities, and the recruitment of patients is therefore different. For example, Hospital Marie Lannelongue does not have an emergency department. It was not impacted in the same way during the 1st wave, since 635 patients were hospitalized at Hospital Paris Saint Joseph compared to 199 at Hospital Marie Lannelongue. Therefore, one would have thought that the prevalence of anxiety, depression and PTSD symptoms among employees would be different in the two hospitals. |
| Importantly, items investigating mental health status before the COVID19 pandemic are not precise, some of them are ambiguous, and we cannot make any solid conclusion about mental health during the pandemic or how prior experience affected current mental health status. | We agree with this comment, and we notified it in the discussion, line 341: *Last, this cross-sectional study cannot demonstrate that COVID-19 is responsible for additional psychological burden in frontline healthcare professionals, as no pre- COVID data are available* |
| Thus, the main aim of the study worded as assessing the psychological impact of the COVID19 pandemic is not feasible at all – the study is cross-sectional and even more, items assessing mental health before the pandemic do not allow us to make some conclusions about it. | As the title of the article indicates, the main objective of the study was to identify factors associated with the mental status of hospital workers. We have modified the last sentence of the introduction to make clear that this objective is the main one. The short title has been also modified |
| I strongly advise changing the analytical strategy. Running so many analyses ended up in having findings compartmentalized, fragmented, and we are missing the big picture about the mental health outcomes in healthcare workers. I suggest running a model in which variables would be included simultaneously to allow for a comprehensible overview of the study variables and their relationships. | We agree with the reviewer that there are many variables. Thus, as suggested for statistical problem we suppressed some of them and we grouped categories (e.g. age is now binarized as compared with 6 categories in the previous version of the paper). Our strategy was based on the strategy published by (Gazmararian et al. 2000) using a classification variables: demographical, professional and emotional. |
| Tables are huge and not very user-friendly – try to present data in a more intelligible fashion. Text describing tables is very detailed and redundant | We simplified the tables by regrouping categories for some variables and suppressing redundant ones. We also simplified text to avoid any redundancy between the results chapter and tables.  Finally, we suppressed table 2. |
| There are some paragraphs in the discussion section that appear to be disconnected from the rest of the text. For example, the third paragraph on p. 23 “So it’s suggested that concrete measures to develop rest rooms and the possibility of having leisure and moments of relaxation are more appropriate to the needs of caregivers than formal psychological support(22,36), and informal mechanisms might be more successful(37) where, for example, counsellors or retired nurses visit healthcare workers in rest areas(25).” How is this paragraph related to the rest of the discussion? | We agree partly with the reviewer. The text is not directly connected to our results. It is more adapted to a perspective section, where we moved it. |
| Language needs proofreading, please check the manuscript carefully – some grammar errors are present in the manuscript | The manuscript has been carefully checked to correct grammar errors |
| Overall, due to a large number of issues in the questionnaire used to collect data that I listed above, I cannot recommend this paper for publishing | We respect the opinion of the reviewer. However, our goal was to identify factors predictive of mental health impairment at the end of the first wave. We do think that our results obtain with many variables offer perspectives for prevention choices during subsequent waves. |

Table 1 : VIF for each regression logistic for symptoms of anxiety

|  | Model 1 | Model 2 |
| --- | --- | --- |
| Age | 1.07 | 1.07 |
| Gender | 1.03 | 1.04 |
| Profession | 1.11 | 1.20 |
| Place of professional practice | 1.03 | 1.04 |
| Respondents who were infected or having infected colleagues or relatives | 1.02 | 1.04 |
| Professional burn-out or depression history | 1.01 | 1.04 |
| Anxiety during 1st Wave |  | 1.11 |
| Fear of contaminating relatives during the 1st wave |  | 1.13 |
| Current job satisfaction |  | 1.06 |

Table 2 : VIF for each regression logistic for symptoms of depression

|  | Model 1 | Model 2 |
| --- | --- | --- |
| Age | 1.06 | 1.07 |
| Gender | 1.02 | 1.04 |
| Place of professional practice | 1.04 | 1.04 |
| Remote work | 1.09 | 1.09 |
| COVID-Unit assignment | 1.13 | 1.13 |
| Marital situation | 1.03 | 1.03 |
| Respondents who were infected or having infected colleagues or relatives | 1.04 | 1.03 |
| Professional burn-out or depression history | 1.02 | 1.03 |
| Anxiety during 1st Wave |  | 1.03 |
| Current job satisfaction |  | 1.03 |

Table 3 : VIF for each regression logistic for symptoms of post-traumatic stress

|  | Model 1 | Model 2 |
| --- | --- | --- |
| Age | 1.04 | 1.05 |
| Gender | 1.02 | 1.03 |
| Place of professional practice | 1.05 | 1.05 |
| Non-clinical professional activity | 1.02 | 1.04 |
| Marital situation | 1.03 | 1.04 |
| Respondents who were infected or having infected colleagues or relatives | 1.02 | 1.03 |
| Professional burn-out or depression history | 1.02 | 1.04 |
| Anxiety during 1st Wave |  | 1.05 |
| Fear of contaminating relatives during the 1st wave |  | 1.05 |
| Current job satisfaction |  | 1.01 |
