# Supplementary material for: Factors associated with psychological symptoms in hospital workers of a French hospital during the COVID-19 pandemic: Lessons from the first wave
Source: PLoS One. 2022 Apr 28;17(4):e0267032. doi: 10.1371/journal.pone.0267032 (PMC9049512; doi:10.1371/journal.pone.0267032)
Supplement: Attachment — Submitted filename: Response to reviewers.docx [file pone.0267032.s005.docx]

Dear Sir,

Thank you for your time and consideration.

We have worked to modify the text according to your recommendations; the manuscript has been thoroughly re-read. We believe the document is more understandable than when first submitted.

We hope that this corrected manuscript is now suitable for publication in Plos One.

In the following paragraphs, we have detailed all the corrections made and added some responses to the reviewers' comments.

Kind regards,

Dr Marguerite d’Ussel and the other authors

| **Comments Editor** | **Answers** |
| --- | --- |
| Scientific reports should be simple and easy to understand, sentences more precise, without ambiguities. | We have revised the entire text to make it more clear, readable and unambiguous |
| After removing adjusted coefficients you still preserved the same table descriptions (did not remove word "adjusted"). Please, be careful with such things. | We try to make more explicit the statistical methods paragraph.  We also modified legend of tables 2, 3 and 4. |
| However, the central issue related to your overall findings is the following: you neither obtained higher prevalence of the studied symptoms during Covid19 pandemics (when compared to pre-pandemic conditions, e.g. Hardy et al., 2019), nor you obtained the difference in these symptoms between those working in Covid-19 units and other units. So, in the light of what you have reported, it is closer to the truth that none of the professional categories were affected by the pandemic, than that everyone was affected "not just front-line or even clinical staff" (p. 26). | That’s true; our study doesn’t allow us to know if the first wave has impacted the hospital workers emotionally.  But we can observe that the level of mental suffering is the same whatever the professional category and the proximity of involvement with patients and their families during the 1^st^ wave.  So we have changed the conclusion in order to be more precise. |
| In other words, the relationships that you have obtain between your predictors and symptoms, mostly reveal the usual pattern of symptom correlates independent of the pandemic. | Exact. And that is interesting according to us! One could have imagined that the frontline workers had higher levels of mental suffering after the 1^st^ wave; our study doesn’t show that.  We have modified the discussion to emphasize that notion |
| Even what appears as something uniquely related to the pandemics - the level of anxiety in the first wave - can reflect nothing more than individual differences in trait anxiety of the participants (especially having in mind that is was measured retrospectively and very roughly with one dichotomous question). You mentioned it sporadically in limitations, but you should make it more salient to a reader. I would recommend to devote full attention to this issue and to elaborate on it properly. | We agree with your remark and we added a section about this notion in the discussion:  One factor which was independently associated with higher symptoms of anxiety, depression and PTSD was having had a personal experience of anxiety during the first wave. But it’s difficult to conclude with this observation; the statistical result is interesting and it could be a track of further prospective work to implement strategies of secondary prevention among the most anxious workers during the crisis. On the other hand our retrospectively measure, with a dichotomy question, is not sufficient to conclude about the relation between anxiety during the event and occurrence of distant emotional symptoms. |
|  |  |
| **Comments Reviewer 1** | **Answers** |
| Reviewer #1: The topic of the manuscript is relevant and actual. The authors have adequately addressed r comments of reviewers, so I believe that the manuscript is now acceptable for publication. There still are some minor suggestions: In the abstract, in 32 line, the authors have written Post-traumatic Stress Distress (PTSD) instead Post-traumatic Stress Disorder | Done |
| The subtitle Materials and Methods, line 94- It would be recommended to stay only Methods (without Materials) | Done |
| **Comments Reviewer 2** | **Answers** |
| I have reviewed the previous version of the paper “Factors associated with psychological symptoms in hospital workers of a French hospital during the COVID-19 pandemic: lessons from the first wave”. The authors have improved the manuscript, and I would like to thank them for that. However, in their letter with responses they listed responses to the comments without including original comments of reviewers, making it very difficult to say which response correspond to which comment. Therefore, I suggest authors to include both comments of the reviewers and responses in the same file. | We are sorry that you didn’t access to the adequate version of our responses  The file called “point-by-point response to reviewer” included a table with the comments in the colon 1 and our responses in the colon 2. We will be attentive that you can access to this form for the second rewieving. |
| Despite manuscript being improved, there are still numerous issues that have to be solved  The most important thing relates to the writing itself – numerous sentences are ambiguous and not clear, several are imprecise, and this must be resolved  This makes manuscript very difficult to read, and impedes making judgements about the results | We edited the manuscript a second time to make it more precise and to avoid any ambiguity. |
| Abstract – “Significant symptoms of anxiety (41%), depression (21%) and PTSD were reported by 41%, 21% and 14% of the participants, respectively” – not clear why these percentages repeat? | Correction:  Significant symptoms of anxiety, depression and PTSD were reported by 41%, 21% and 14% of the participants, respectively. |
| Abstract – “There was no difference regarding type of occupation or assignment in a COVID-19 unit.” – there was no difference in what? | There was no difference among workers in anxiety, depression and PTSD symptoms regarding type of occupation or assignment in a COVID-19 unit. |
| p. 10 – “Compared with female gender, male gender was associated with a significantly lower prevalence of symptoms of anxiety (87% vs. 13%; P=0.001) and PTSD (89% vs. 11%; 178 P=0.029), but a comparable prevalence of depression (83% vs. 17%; P=0.73). “– check percentages for depression | We have verified the figures. There is no mistake in the tables. But inappropriate choice of figures was done in the text of results section. This choice does not change the interpretation of the p-value. We corrected it. |
| p. 10 – “Medical professionals had lower prevalence of anxiety symptoms than other workers (11%, P=0.033).” – lower prevalence than other workers, how are these other workers? Sentence is not clear. | We compared the different professional categorizations by regrouping different similar professions in a same group, to limit the number of variables: medical professionals (physician, pharmacologist, biologist), administrative healthcare workers, caregivers (nurse, assistant nurse, nurse manager), others caregivers (Physiotherapist, stretcher-bearer, Radiologic Technologist, Psychologist), midwifes and others workers.  The reviewer is right, we clarified the sentence.  (line 150, table 1) |
| p. 10 – “Healthcare workers not working in a COVID-19 Unit were more prone to depression (60% vs. 40%; P=0.023)” – more prone to depression compared to whom? | Tables are correct. Inappropriate choice of figures was done in the text of results section. This choice does not change the interpretation of the p-value. We corrected the results section. |
| p. 16 – “Regarding emotional experience at the time of the first wave, the hospital employees who reported to have been anxious during the first wave had more anxiety (86% vs. 14%; p<0.001)more depression (76% vs. 24%; P<0.001) and more PTSD symptoms (90% vs. 10%; P<0.001) in post crisis than those who have not reported anxiety.” – what is post crisis , sentence is not clear. | Tables are correct. Inappropriate choice of figures was done in the text of results section. This choice does not change the interpretation of the p-value. We corrected the results section. |
| p. 16 – “Compared to patients without history 211 of burnout or depression those having such a history had greater anxiety (34% vs. 66%; P<0.001) , depression (61% vs. 39%; P<0.001), and PTSD symptoms (34% vs. 66%; P=0.001).” – please be consistent, either write bigger vs smaller numbers, or smaller vs bigger | Tables are correct. Inappropriate choice of figures was done in the text of results section. This choice does not change the interpretation of the p-value. We corrected the results section. |
| p. 17 – “In multivariable analysis (tables 2, 3 and 4), healthcare workers who reported to have been anxious during the first wave reported more anxiety (OR, 5.83; 95% CI: 3.89-8.90), depression (OR, 1.70; 95% CI: 1.11–2.65) and PTSD symptoms (OR, 4.47; 95% CI: 2.35–9.27)” – it seems that something is wrong with this statement – those who said that are anxious reported they were more anxious – it’s circular, check this. | We note that this notion of anxiety during the 1^st^ wave is not clear and we have specified it in the entire document:  In multivariable analysis (tables 2, 3 and 4), healthcare workers who retrospectively reported that they felt anxious during the 1st wave had more symptoms of anxiety (OR, 5.83; 95% CI: 3.89-8.90), depression (OR, 1.70; 95% CI: 1.11–2.65) and PTSD symptoms (OR, 4.47; 95% CI: 2.35–9.27) at the moment of the survey. |
| p. 21 – “It appears to be a strong relationship 318 between this emotional experience at the time of the first wave and the prevalence of significant symptoms of anxiety, depression and PTSD afterwards.” Perhaps I’m missing something, but this sentence is not clear to me – the paper investigates mental health in the first wave of the pandemic, so I don’t understand to what “significant symptoms of anxiety, depression and PTSD afterwards” refer? | Our survey had been diffused around 3 months after the end of the 1^st^ wave in our hospital located in Paris, without knowing that other waves would come later.  We wanted to investigate the emotional status of the hospital workers after this event that was the 1^st^ wave.  Our study shows there is a relationship between those who retrospectively felt anxious during the 1^st^ wave, and the prevalence of symptoms of anxiety, depression and PTSD some weeks later:  *It appears to be a strong relationship between this anxious experience just at the time of the first wave (reported retrospectively by the responders) and the prevalence of significant symptoms of anxiety, depression and PTSD afterwards.* |
| p. 21 – “The fact that introducing this variable in the model resulted in rendering some predictive variables (e.g. gender) non-significant strongly suggests that anxiety at the time of the first wave explains a substantial part of the association between vulnerability factors (e.g. history of depression) and subsequent symptoms of anxiety, depression and PTSD” – again, I don’t understand this sentence. What is the variable introduced in the model? ‘what is the connection between gender, history of depression, and anxiety during the first wave of the pandemics? And how this information informs us about secondary prevention strategies? The study explores mental health issues after the first wave, but throughout the text authors compare mental health before the first wave, during the first wave and after the first wave. This creates confusion as there are no data to compare mental health during and after the first wave. | We have totally re-written this section in order to let it more readable:  By using a hierarchical strategy in the multivariable analysis, we observed the role played by the anxious experience on the studied variables: the risk of symptoms of anxiety was higher among women in the model 1, but if we enter the emotional experience as variable in the model 2, symptoms of anxiety and gender were no longer associated. Thus, although woman gender is associated with symptoms of anxiety, the causal pathway for these relation is likely to be through the emotional experience during the crisis.  We found similar results for participants with a history of burnout or depression who had more PTSD symptoms: when we adjusted with the variables of emotional experience during the crisis, history of burnout or depression was no longer related to PTSD symptoms. The relationship between history of burnout or depression and PTSD appears to be mediated by the emotional experience of the crisis.  The impact of the personal experience of anxiety during the crisis was particularly strong, especially when the respondents said that this anxiety was focused on their job. There is a strong relationship between this anxious experience just at the time of the first wave (reported retrospectively by the responders) and the risk of symptoms of anxiety, symptoms of depression and PTSD afterwards. So we can consider that identifying that staffs who feel most anxious during a health crisis and implementing secondary prevention strategies targeted at these staff, we could reduce the prevalence of post-crisis mental health disorders. |
| Table 1 – number of participants responding to specific items differs, and it would be good to include this information consistently for each item – at the moment, for some items information is given, for some it is omitted. | When the number of participants was 780 (total), we didn’t notified it; but if you think is clearer we notified for each item |
| Table 2 and Table 3 – explain acronyms in the title, HADa and HADd in the notes. | done |
| Table 3 – check cell Place of professional practice, duplicated text | done |
| Please don’t start sentences with numbers (percentages). | done |
| To conclude, the major issue preventing me from recommending the manuscript for publication is the writing which has to be substantially improved. |  |
