# Supplementary material for: Factors associated with psychological symptoms in hospital workers of a French hospital during the COVID-19 pandemic: Lessons from the first wave
Source: PLoS One. 2022 Apr 28;17(4):e0267032. doi: 10.1371/journal.pone.0267032 (PMC9049512; doi:10.1371/journal.pone.0267032)
Supplement: Attachment — Submitted filename: Response to reviewers.docx [file pone.0267032.s006.docx]

Dear Sir,

Thank you for your time and consideration.

The manuscript has been corrected by a professional translator and we made many modifications in the text in order to let it less ambiguous. Especially, we clearly indicated that the cross-sectional nature of the data limits our ability to assess whether there might be causal relationship between the health crisis and mental health outcomes, as no pre-COVID data are available. We added it in the conclusion as you encouraged us to do.

We hope that this corrected manuscript is now suitable for publication in Plos One.

In the following paragraphs, we have detailed all the corrections made and added some responses to the reviewers' comments.

Kind regards,

Dr Marguerite d’Ussel and the other authors

| **Comments Editor** | **Answers** |
| --- | --- |
| 1. I would like you to take suggestion on improvement of the language, by r#2, seriously. It means that you have to have proofread done by the professional translator. Without the substantial improvement of the readability of the text, we will not be able to accept the manuscript. The reason is that, generally speaking, your sentences are ambiguous and, frequently, difficult to understand. | The manuscript has been entirely corrected by a professional translator. |
| 2. It also seems to me that you did not give due consideration to my comments regarding the way you generally interpreted your results. To reiterate, my understanding is that your overall findings are in accordance with the following scenario: the crisis did not affect the level of symptoms you assessed in health care workers in any substantial manner (namely the prevalence of the symptoms among health care workers did not seem to be higher compared to pre-pandemic conditions); as a consequence, the differences between subgroups of health care workers were not found; Obviously, the crucial feature of such a scenario is that it is unrelated to pandemics. You have address this issue in the limitations paragraph (rows 425-430), but I urge you to mention it in conclusions. For example, you can add the first sentence in Conclusions something like...but it does not appear to be elevated significantly compared to the the pre-pandemic conditions. | We modified every sentence in the manuscript which could still suggest that there was a causal relationship between the pandemic and mental health outcomes.  We added it clearly in conclusion. |
| relationships between anxiety during the first wave (retrospectively measured) and depression and anxiety could result from the simple fact that people with the higher level of trait anxiety are prone to be more anxious in the past, as well as to the tendency of the people with higher trait anxiety to retrospectively report higher level of anxiety (even if they did not have the level of anxiety they tend to report, which is the limitation of the cross-sectional nature of the study). | That’s true; and we wrote it clearly in discussion (line 312) |
| **Comments Reviewer 2** | **Answers** |
| The major drawback of this manuscript is the quality of the language. I understand that the authors tried to improve the manuscript, but it is of absolute importance that the paper be rewritten by a professional (native speaker of the English language). It is not possible to list all problems I noticed, and I flagged only some. If the text is not edited and proofread, I cannot recommend its publication. I understand this feedback is disappointing but my intention is only to make the manuscript better, and I hope that authors will interpret my feedback positively.  Below I list some of the language issues I detected. | We understood that major obstacle and a professional translator has proofread the entire manuscript.  We thank the reviewer for having listed the major issues and suggested corrections |
| Lines 106-107: The entire hospital workers were invited to complete a survey online, with a link sent to the mailing list.  Instead of ENTIRE it should be ALL. | Done |
| Lines 107-108: This was a questionnaire in "Microsoft forms" electronic format that allows the secure sending and storage of responses.  “THIS” is not clear. I understand what you wanted to say, but it is not grammatically correct. Rewrite the sentence. | Done |
| Lines 108-109: Inclusion criteria were for the responders to have worked during the period from March 15 to May 15 and to be a volunteer to complete the survey.  This sentence has to be rephrased, wording is odd. | Done |
| Lines 114-115: They were: (the whole of the submitted questionnaire is accessible as supporting information 115 S1 Appendix).  Again, language is not okay. | Done |
| Lines 130-131: It was made clear in the questionnaire that the stressful event to which respondents were referring was the health crisis (considered to have been over a month old at the time of the survey).  “over a month old” – not clear to what you refer. This has to be revised. | Done |
| Lines 170-171: Half of the participants (46%) reported having taken care of patients with COVID-19 frequently (every worked day) or regularly (at least one day a week).  Every working day, not worked day. Also, one day in a week, or once a week. | Done |
| Lines 171-172: 60% reported that a colleague, a 172 friend, or close relative had been infected by SARS-CoV-2.  The sentence should not start with a number. | Done |
| Lines 174-176: Among the hospital employees, 62% reported that they were anxious during the period of 1st wave: 86% for their 176 family, 57% for themselves, 51% at work and 42% for the others.  The sentence is not clear. The colon makes it very unclear, how were these percentages obtained. Revise the sentence. | Done |
| Lines 189-190: This proportion was comparable in the presence or absence of symptoms of depression (17% vs 19%; p=0.73).  I don’t understand this sentence. | Done |
| Lines 248-254: There were more hospital employees who retrospectively reported at the moment of the survey that the first wave made them anxious in the group with symptoms of anxiety than in the group without symptoms of anxiety (86% vs 46%; p = 0.001), the same was true for symptoms of depression (76% vs 59%; p<0.001). There were more employees who reported a history of burnout or depression in the group with symptoms of anxiety than in the group without symptoms of anxiety (34% vs 15%; p < 0.001), the same was true for symptoms of depression (39% vs 18%; p < 0.001), and PTSD (34% vs 21%; p=0.001).  These sentences are not clear. ? Also, they appear to be circular – more people with anxiety symptoms is in the group with symptom of anxiety. Where there more people with symptoms of depression in the group of people with anxiety or in the group with depression? Second sentence is also not clear to me – what was true for the symptoms of depression and PTSD? | Done |
| Lines 357-359: But it’s difficult to conclude with this observation; the statistical result is interesting and it could be a track of further prospective work to implement strategies of secondary prevention among the most anxious workers during the crisis.  OBSERVATION is not the appropriate word. Perhaps you can say: We cannot made final conclusion based on results of one study, … | Done |
